# Baseline quality of life as a prognostic survival tool in patients receiving sunitinib for metastatic renal cell carcinoma

**DOI:** 10.1038/bjc.2011.589

**Published:** 2012-01-12

**Authors:** D Cella, A G Bushmakin, J C Cappelleri, C Charbonneau, M D Michaelson, R J Motzer

**Affiliations:** 1 Department of Medical Social Sciences, Northwestern University Feinberg School of Medicine, Chicago, IL, USA; 2 Global Research and Development, Pfizer Oncology, New London, CT, USA; 3 Global Outcomes Research, Pfizer Oncology, New York, NY, USA; 4 Massachusetts General Hospital Cancer Center, Boston, MA, USA; 5 Memorial Sloan-Kettering Cancer Center, New York, NY, USA

**Keywords:** metastatic renal cell carcinoma, quality of life, prognostic tool, sunitinib, survival, Weibull model

## Abstract

**Background:**

In a randomized phase III trial of sunitinib *vs* interferon-alfa (IFN-*α*) in metastatic renal cell carcinoma (mRCC), better baseline quality of life (QoL) was predictive of longer survival. Using this dataset, we have developed a novel prognostic tool that establishes a relationship between baseline QoL scores and median survival time.

**Methods:**

Baseline QoL was assessed using the FACT-Kidney Symptom Index-15 item (FKSI-15), its disease-related symptoms (FKSI-DRS) subscale, and the Functional Assessment of Cancer Therapy–General (FACT-G) scale. Weibull models estimated median progression-free survival (mPFS) and overall survival (mOS) as a function of baseline QoL.

**Results:**

Longer PFS and OS were associated with higher baseline FKSI-15, FKSI-DRS, and FACT-G scores (*P*<0.05), and baseline FKSI-15 score was the best predictor of survival. For example, for a baseline FKSI-15 score of 60, the predicted mPFS was 67.9 weeks, and predicted mOS was 240.6 weeks. The magnitude of benefit was greater with sunitinib *vs* IFN-*α* for a given baseline QoL score.

**Conclusion:**

This novel tool indicates that baseline FKSI-15 scores were linked to mPFS and mOS in a clear and interpretable way. The results support evaluation of patient-reported QoL symptoms at baseline as a prognostic indicator of survival in clinical research and practice.

Historically, metastatic renal cell carcinoma (mRCC) has been a difficult disease to manage, because of its resistance to chemotherapy and radiotherapy. The development of targeted therapies (such as small molecule kinase inhibitors and vascular endothelial growth factor (VEGF) antibodies) has led to more promising clinical outcomes (Sun *et al*, 2010). Sunitinib malate (SUTENT; Pfizer Inc., New York, NY, USA) is an oral multitargeted inhibitor of VEGF receptor, platelet-derived growth factor receptor, and several other kinases that is approved for the treatment of advanced RCC as well as imatinib-resistant/intolerant gastrointestinal stromal tumour (Chow and Eckhardt, 2007). In a randomized, multicenter, phase III trial (ClinicalTrials.gov: NCT00083889; sponsor: Pfizer), sunitinib showed superior progression-free survival (PFS; the primary endpoint) over interferon-alfa (IFN-*α*) as first-line mRCC therapy (11 *vs* 5 months (*P*<0.001)); in addition, median overall survival (mOS) with sunitinib was >2 years (26.4 *vs* 21.8 months with IFN-*α* (*P*=0.051)) (Motzer *et al*, 2009).

Various clinical factors – such as haematological and inflammatory markers, site and number of metastases, performance status, tumour stage, time between diagnosis and treatment, and previous surgery – have been investigated to determine their ability to predict survival in patients with mRCC (Motzer *et al*, 2002, 2004; Bensalah *et al*, 2006; Suppiah *et al*, 2006; Choueiri *et al*, 2007; Kwak *et al*, 2007; Patil *et al*, 2011). Some of these factors have been used to categorize patients into risk groups to aid clinical trial design and the tailoring of treatment strategies to optimize outcomes. However, few studies have evaluated baseline quality of life (QoL) as a predictor of survival.

Using interim data from the sunitinib phase III trial mentioned above, we previously showed that higher baseline QoL scores were associated with improved PFS (Cella *et al*, 2009). Using final data from the sunitinib phase III trial, we have now developed a novel prognostic tool that converts baseline QoL scores into median duration of PFS and OS in patients treated with sunitinib.

## Patients and Methods

### Patients and study design

Full details have been described previously (Motzer *et al*, 2007, 2009).

This phase III study population comprised 750 patients ⩾18 years with histologically confirmed mRCC with a component of clear-cell histology. Key eligibility criteria included: no previous systemic therapy for RCC; measurable disease, Eastern Cooperative Oncology Group performance status 0 or 1; and adequate hepatic, renal, and cardiac function. All patients provided written informed consent.

Patients were randomly assigned in a 1 : 1 ratio to receive either sunitinib or IFN-*α* in repeated 6-week cycles. Sunitinib was administered orally at 50 mg day for 4 weeks, followed by 2 weeks off treatment (Schedule 4/2). IFN-*α* was administered as a subcutaneous injection on three non-consecutive days per week, starting at 3 million units (MU) for the first week, 6 MU for the second week, and 9 MU thereafter.

The study was approved by the institutional review board or ethics committee at participating centres and was conducted in accordance with provisions of the Declaration of Helsinki and Good Clinical Practice guidelines.

### Assessments

As described previously (Motzer *et al*, 2007, 2009), tumour imaging was performed at screening, on day 28 of cycles 1–4 and even cycles thereafter, whenever progression was suspected or to confirm response, and at the end of treatment. Tumour response was assessed by investigators according to Response Evaluation Criteria in Solid Tumours (Therasse *et al*, 2000). PFS was defined as the time from randomization to first documentation of objective tumour progression or death due to any cause. Patients were followed off-study every 2 months for survival.

**Figure 1:**
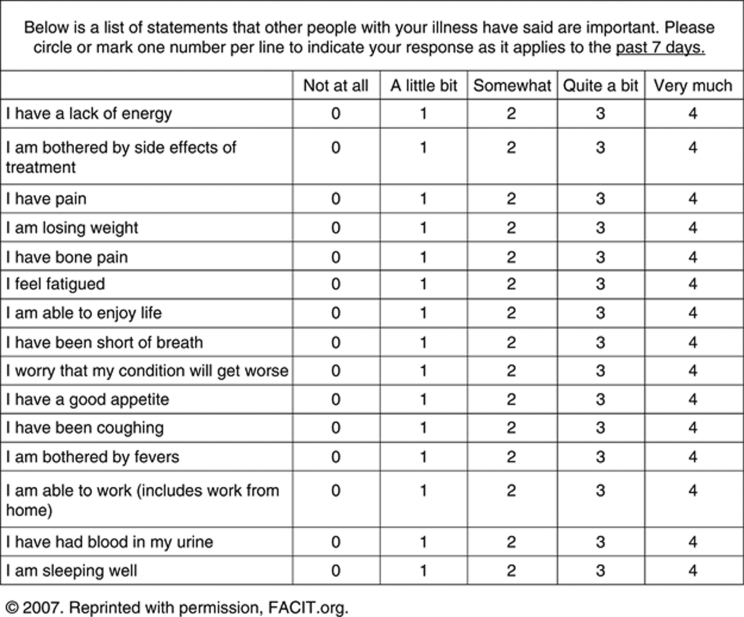
The Functional Assessment of Cancer Therapy–Kidney Symptom Index (FKSI)-15 item long form questionnaire 2007. Reprinted with permission, FACIT.org.

QoL was measured at baseline using all available data from the following patient-reported questionnaires: the Functional Assessment of Cancer Therapy–Kidney Symptom Index (FKSI)-15 item (Figure 1) (Cella *et al*, 2006), which measures symptoms related to kidney cancer such as ‘I feel fatigued’, ‘I have been short of breath’, ‘I am bothered by fevers’, ‘I have had blood in my urine’, and rates the severity of each item; its nine-item disease-related symptoms (FKSI-DRS) subscale (Cella *et al*, 2007); the Functional Assessment of Cancer Therapy–General (FACT-G) (Cella *et al*, 1993); and its four subscales (physical well-being, social/family well-being, emotional well-being, and functional well-being). Higher scores indicated better outcomes (better QoL or fewer symptoms).

### Statistical methods and analysis

Weibull (parametric) models were used to establish a relationship between baseline QoL score and median survival time (using SAS LIFEREG (Kleinbaum and Klein, 2005; SAS Institute Inc., 2008)). These models were applied separately to the sunitinib and IFN-*α* arms, and OS and PFS were analysed as separate outcomes. In order to estimate 95% confidence intervals (CIs) for the between-treatment differences in estimated median survival times, 50 000 bootstrap simulations were performed. Akaike's information criterion (AIC) (Akaike, 1974), a measure of goodness of fit, where lower values indicate a better fit, was used to identify the QoL instrument that provided the best predictive power for median survival time. Additionally, a Kaplan–Meier estimation method (Kaplan and Meier, 1958) (non-parametric approach) was used to perform sensitivity analyses by forming, for each QoL measure, three tertile groups on QoL scores of approximately equal size (the lowest, highest, and in-between scores), and estimating for each group the median OS time and median PFS time, as well as by examining the entire Kaplan–Meier curve for each group across the QoL scores (using SAS PROC LIFETEST (SAS Institute Inc., 2008)).

## Results

### Baseline characteristics

As previously reported (Cella *et al*, 2008, 2009, 2010), there were no significant between-treatment differences in the baseline characteristics of patients – including baseline QoL scores, which were in the moderate range (Table 1).

For example, baseline FKSI-DRS scores (mean±s.d.) were 29.74±5.24 and 29.55±5.03 for patients in the sunitinib and IFN-*α* arms, respectively (FKSI-DRS scores can range from 0 (most severe symptoms) to 36 (no symptoms)).

### Predictive value of baseline QoL

All available data for the FACT-Kidney Symptom Index-15 item (FKSI-15), FKSI-DRS, and FACT-G at baseline were used in the analyses. Longer median PFS and OS were associated with higher (more favourable) baseline FKSI-15, FKSI-DRS, and FACT-G scores (each *P*<0.05 from the Weibull model) in patients on sunitinib or IFN-*α*.

Based on it having the lowest AIC value overall (range for OS models: from 792.3 to 826.0; range for PFS models: from 841.2 to 851.3), the baseline FKSI-15 score was the best QoL instrument for predicting PFS and OS.

**Figure 2:**
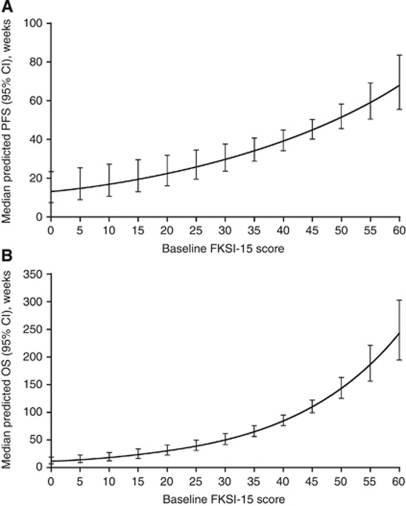
Predicted median (**A**) PFS and (**B**) OS as a function of baseline FKSI-15 scores in patients on sunitinib. Abbreviations: CI=confidence interval; FKSI-15=Functional Assessment of Cancer Therapy–Kidney Symptom Index–15 item; OS=overall survival; PFS=progression-free survival.

Figure 2 and Table 2 show predicted median PFS and median OS as a function of baseline FKSI-15 scores in patients on sunitinib. Table 3 lists the estimated parameters of the models for PFS and OS. For baseline FKSI-15 scores of 30 and 60 (where 0=most symptoms and 60=no symptoms), median PFS was predicted to be 29.6 and 67.9 weeks, respectively, and median OS was predicted to be 49.3 and 240.6 weeks, respectively. Therefore, relative to patients with lower baseline (less favourable) FKSI-15 scores, patients with higher FKSI-15 scores at baseline (i.e., those who had fewest cancer-related symptoms) experienced longer median PFS and median OS, which increased exponentially after a baseline FKSI-15 score of around 30.

### Between-treatment comparisons

**Figure 3:**
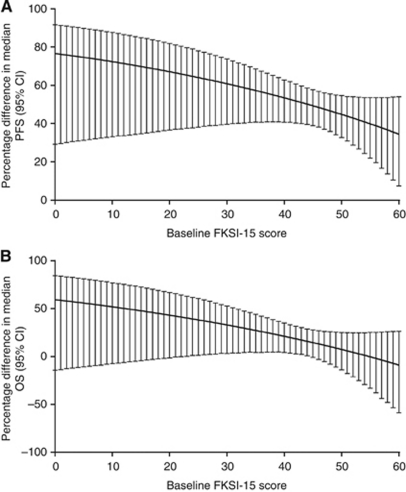
Between-treatment (sunitinib *vs* IFN-*α*) percentage differences in predicted median (**A**) PFS and (**B**) OS as a function of baseline FKSI-15 scores. Abbreviations: PFS=progression-free survival; OS=overall survival; CI=confidence interval; FKSI-15=Functional Assessment of Cancer Therapy–Kidney Symptom Index–15 item. Note: a two-sided 95% CI for the between-treatment difference that does not contain 0 indicates that the difference in the treatment arms was statistically significant.

A comparison of the between-treatment (sunitinib *vs* IFN-*α*) percentage differences in predicted median PFS and (separately) median OS as a function of baseline FKSI-15 scores indicated that, for a given baseline QoL score, the magnitude of benefit was greater with sunitinib relative to IFN-*α* (Figure 3). For the same FKSI-15 score, predicted median PFS was always significantly better in the sunitinib arm relative to the IFN-*α* arm; predicted median OS trended in favour of sunitinib, but not all the between-treatment differences were significant.

In addition, the survival benefit of sunitinib relative to IFN-*α* increased with worsening baseline kidney symptoms (lower FKSI-15 scores); Figure 3A shows that a patient on sunitinib with a baseline FKSI-15 score in the range 0–20, for example, had a predicted median PFS that was ∼70% longer than that of a patient on IFN-*α* with the same baseline FKSI-15 score range; whereas, a patient on sunitinib with a baseline FKSI-15 score in the range 50–60 had a predicted median PFS that was ∼40% longer than that of the equivalent IFN-*α* patient. All between-treatment percentage differences in predicted median PFS were statistically significant, based on the two-sided 95% CIs not containing 0.

Similarly, there was an ∼50% difference in predicted median OS, favouring sunitinib, in patients with baseline FKSI-15 scores in the range 0–22, although this difference was not statistically significant (Figure 3B). A patient on sunitinib with a baseline FKSI-15 score in the range 23–44 had a predicted median OS that was∼30% longer than that of the equivalent IFN-*α* patient, and statistically significant (Figure 3B). In patients with baseline FKSI-15 scores in the range 45–60, between-treatment percentage differences in predicted median OS were small and not statistically significant (Figure 3B).

### Sensitivity analyses

Sensitivity analyses using Kaplan–Meier (non-parametric) estimation supported the results of the parametric modelling. Differences between the two models in terms of predicted median PFS, as well as median OS, were less than 10% (data not shown).

## Discussion

The randomized phase III trial of first-line sunitinib in mRCC patients showed superior PFS *vs* IFN-*α* (11 *vs* 5 months (*P*<0.001)), and median OS of more than 2 years (26.4 *vs* 21.8 months with IFN-*α* (*P*=0.051) (Motzer *et al*, 2009)). In a previous publication, further analysis of interim data from this trial showed that baseline QoL (including FKSI-15 scores) was a significant predictor of PFS, even after other prognostic variables (e.g., treatment and key demographic and clinical variables) were added to the model (Cella *et al*, 2009). In this full (final) dataset, QoL continues to be a predictor of PFS and also OS, even after other prognostic variables were added to the model (age, sex, baseline Eastern Cooperative Oncology Group performance status, number of Memorial Sloan–Kettering Cancer Center risk factors, prior nephrectomy and radiotherapy, number of metastases, and sites of metastases).

In the present report, we describe a novel prognostic tool that establishes a relationship between baseline QoL and both median PFS and OS. The question posed in this research is whether – and to what extent – baseline FKSI-15 scores predict median survival, not which of several prognostic factors *best* predict median survival. As such, this effort extends beyond prior research. The current study adds originality by predicting median PFS and OS times and their 95% CI, from the baseline scores. As such, the analyses take into account the inherent variability in median PFS or OS for any given score.

Our results revealed a robust relationship between baseline FKSI-15 scores and median survival time. Increased median PFS and OS were associated with higher (better or more favourable; fewer symptoms) baseline FKSI-15 scores in patients on sunitinib or IFN-*α*. These findings are consistent with our earlier analysis (Cella *et al*, 2009) and with a phase III trial of second-line sorafenib in mRCC patients, which showed that the total baseline FKSI score was predictive for OS (*P*<0.0001) and that favourable baseline QoL scores were associated with subsequent improvement in survival outcomes (Bukowski *et al*, 2007). That the 15-item FKSI questionnaire was the best predictor of PFS and OS likely relates to the fact that this instrument is specific to symptoms of kidney cancer (unlike the FACT-G that provides a more general assessment of QoL) (Cella *et al*, 2006).

Our findings based on parametric modelling are considered to be robust as they were supported by results of the sensitivity analyses with the Kaplan–Meier (non-parametric) estimation. Although the Weibull parametric model used in this analysis is not a novel analytic approach in itself, our application of the model to associate a given baseline score with a median survival time is original. Similarly, our application of the Kaplan–Meier estimation method for performing sensitivity analyses is novel in this setting.

The present results also demonstrated that median PFS and OS were superior for sunitinib relative to IFN-*α* for a given patient QoL score. For the same FKSI-15 score, the predicted PFS was always significantly better in the sunitinib arm relative to the IFN-*α* arm, while the predicted OS trended in favor of sunitinib but the between-treatment differences were not always significant.

These findings reaffirm the importance of evaluating patient-reported QoL. For the clinician, knowledge of baseline FKSI-15 scores provides additional guidance on likely outcomes for individual patients, whereas for the patient, it is encouraging to know that by reporting their symptoms they are contributing valuable information that will help to inform decisions on their overall management.

In summary, this novel tool indicates that baseline FKSI-15 scores were linked to median PFS and OS in a clear and interpretable way in these mRCC patients treated with sunitinib or IFN-*α*. The results support the evaluation of patient-reported QoL symptoms at baseline as a prognostic indicator of survival times in clinical trials and practice. Although the modelling approach used in this analysis is applicable to other clinical trials (Trask *et al*, 2011), the findings reported here only pertain to the study population and treatments investigated in the present trial.

## Figures and Tables

**Table 1:** Baseline patient demographics and clinical characteristics (Cella *et al*, 2008, 2009)

	**Sunitinib (*n*=375)**	**IFN-*α* (*n*=375)**
Median age (years)	61	60
		
*Sex, %*		
Male	71	72
Female	29	28
		
*ECOG PS, %* a		
0	62	61
1	38	38
2	0	1
		
*MSKCC risk factors, %*		
0	38	34
1–2	56	59
⩾3	6	7
		
Previous nephrectomy, %	90	89
Previous radiotherapy, %	14	14
		
*No. of disease sites, %*		
1	14	19
2	29	30
⩾3	57	51
		
*Site of metastasis, %*		
Lung	78	79
Liver	26	24
Bone	30	30
Lymph nodes	58	53
		
*QoL scores (mean±s.d.)*		
FKSI-15	46.45±8.46	46.10±8.70
FKSI-DRS	29.74±5.24	29.55±5.03
FACT-G	82.30±15.20	81.25±16.04

Abbreviations: ECOG PS=Eastern Cooperative Oncology Group performance status; FACT-G=Functional Assessment of Cancer Therapy-General; FKSI-15=Functional Assessment of Cancer Therapy–Kidney Symptom Index–15 item; FKSI-DRS=Functional Assessment of Cancer Therapy-Kidney Symptom Index-Disease-Related Symptoms; *IFN*-*α=*interferon-alfa; MSKCC=Memorial Sloan-Kettering Cancer Center; *n*=number of subjects; QoL=quality of life; s.d.=standard deviation.

a All patients had ECOG PS of 0 or 1 at the time eligibility was determined; four patients in the IFN-*α* group had an ECOG PS of 2 on the day of starting study treatment.

**Table 2:** Predicted median PFS and OS by baseline FKSI-15 score in patients on sunitinib

**Baseline FKSI-15 score**	**Predicted PFS (95% CI) *n*=372**	**Predicted OS (95% CI) *n*=372**
0	12.94 (7.19, 23.30)	10.10 (5.89, 17.34)
5	14.86 (8.78, 25.16)	13.16 (8.14, 21.28)
10	17.06 (10.71, 27.18)	17.14 (11.25, 26.11)
15	19.59 (13.06, 29.38)	22.32 (15.53, 32.07)
20	22.49 (15.92, 31.78)	29.06 (21.43, 39.42)
25	25.81 (19.37, 34.41)	37.85 (29.53, 48.53)
30	29.64 (23.53, 37.34)	49.30 (40.56, 59.91)
35	34.03 (28.46, 40.69)	64.20 (55.40, 74.40)
40	39.07 (34.12, 44.75)	83.62 (74.64, 93.67)
45	44.86 (40.08, 50.20)	108.90 (97.83, 121.21)
50	51.50 (45.60, 58.16)	141.82 (124.25, 161.88)
55	59.13 (50.53, 69.20)	184.70 (154.91, 220.23)
60	67.89 (55.24, 83.43)	240.55 (191.57, 302.05)

Abbreviations: CI=confidence interval; FKSI-15=Functional Assessment of Cancer Therapy–Kidney Symptom Index–15 item; OS=overall survival; PFS=progression-free survival.

There were no observed scores below 20 points on the FKSI-15. As such, prediction estimates were extrapolations in this range and may not be as reliable as other prediction estimates where data were observed.

**Table 3:** Parameter estimates for PFS and OS models (*n*=372)

**Parameter**	**Estimate**	**95% CI**
*Intercept*		
PFS	2.86	2.28, 3.44
OS	2.58	2.05, 3.11
		
*Score*		
PFS	0.03	0.02, 0.04
OS	0.05	0.04, 0.07
		
*Scale*		
PFS	0.82	0.73, 0.91
OS	0.74	0.65, 0.84
		
*Weibull shape*		
PFS	1.23	1.10, 1.37
OS	1.36	1.20, 1.54

Abbreviations: CI=confidence interval; OS=overall survival; PFS=progression-free survival.
